# A Bill for Regulating the Profession of Physic and Surgery

**Published:** 1845-04

**Authors:** 


### Art. XVII.

*A Bill for regulating the Profession of Physic and Surgery. Ordered by the House of Commons to be printed 25th Feb.* 1845.

Taking up this Bill in the same point of view as a book, we may regard it as a second edition, ‘revised and corrected,’ of that of which we gave so ample an analysis and criticism in our last Number. Our present business, therefore, is, to notice the alterations that have been made in the work since it was last under review. They are not numerous nor very considerable, though some of them are important, and, we believe we may add, all are improvements. We shall notice the alterations under the same heads, and in the same order, as when we last examined the Bill, referring, under each head, to the page in our last Number where the subject was treated.

I. *Of the Bill in relation to the education of medical men.* (p. 194.)

1. *Medical students.* Although nothing new as to the education of students, is added in a direct form, the clause regulating the registration of students, is altered in such a way as to enable the Council of Health to ascertain, with certainty, where and how long every one has studied. The registration must be annual, and no student will be admitted to examination unless he has been duly registered. The registration fee is very moderate, being only two shillings and sixpence each year. The Apothecaries' act being only partially repealed in the present Bill, the old clause respecting apprenticeships still stands, but as the power of licensing is transferred from the Company to the Council of Health, it becomes a dead letter. It cannot possibly be intended, that the exercise of the *protective power* left by the new Bill with the Company, must depend on the fact of the party having served an apprenticeship!

2. *Licentiates of medicine and surgery.* The only alteration that applies to general practitioners is the institution of an examination in midwifery, which, however, is not made compulsory. The names of persons who have “passed such special examination satisfactorily,” will be distinguished by a mark in the register.

3. *Surgeons.* A most important alteration, on the necessity of which we insisted in our former article, is made in the education of surgeons. They must now undergo the same examination as the licentiates, that is, be examined in medicine.

4. *Physicians.* The only alterations affecting physicians are the following, which are decided improvements: 1. Instead of being obliged to pass “the two years *next before* obtaining their degree” at the university granting it, they may choose *any two* years after being matriculated, to reside at the seat of such university. 2. Physicians obtaining degrees from foreign universities, must now give proof of “residence and study at the seat of one or more universities, during at least *three* years, one of them, at least, being at the university by which the degree is granted.” In the former Bill, one year's residence at an university was alone required.

II. *Of the Bill in relation to the mode of examination of candidates*

*for the licence.* (p. 199.) The alterations under this head are decided improvements, as far as they go; but we greatly regret that Sir James Graham did not fully follow out what he has stated to be his own views; we mean the establishment of a distinct board of examiners unconnected with the colleges, and the obligation on every person entering the profession, to undergo the same primary examination. But, we presume, he found the colleges too untractable. The improvements introduced in the new Bill are: 1, the abolition of the Glasgow examination board; 2, the obliging the Apothecaries' Company to select the examiners sent by them to join the examiners of the College of Physicians, from licentiates of medicine and surgery of ten years' standing, instead of members of the Society of Apothecaries, as formerly.

III. *Of the Bill in relation to the equality of privileges of medical men in practice.* (p. 202.) The only change under this head, is the introduction of a clause to assimilate the privileges of licentiates more closely with those of surgeons and physicians, than was the case in the first Bill. The new clause enacts, "That every person registered after examination as a licentiate in medicine and surgery, shall be admitted as a Member or Licentiate of the Royal College of Surgeons in that part of the United Kingdom in which he shall have received his letters testimonial; and every such person who shall afterwards remove into any other part of the United Kingdom, and shall practise there as a licentiate in Medicine and Surgery, shall be required to enrol himself as a Member or Licentiate of the Royal College of Surgeons of that part of the United Kingdom to which he shall so remove, and in each case shall be entitled to be so admitted and enrolled without further examination, and on payment of the like fees of admission, and on complying with the same conditions as are required of other persons who have passed their examinations and paid their examination-fees for the purpose of being admitted Members or Licentiates of the said Colleges respectively."

The important improvement here is, that henceforth all Licentiates can *claim to be Members of the College of Surgeons,* on passing the licentiate's examinations and paying the fees of admission.

IV. *Of the Bill in relation to the registration of licensed practitioners.* (p. 203.) The only changes under this head are: 1, the improved registration of students already noticed; 2, the explicit declaration that the registration fee is only payable *once*; 3, the empowering all persons then practising or entitled to practise in any *part of the United Kingdom,* at the time of the passing of the Bill, to register, on producing their diplomas and paying the registration-fee,—viz. twenty shillings as a physician or surgeon, and five shillings as a licentiate.

V. *Of the Bill, in relation to the incorporation of the profession.* (p. 204.) The only alteration, under this head is the one already noticed, relating to the admission of licentiates as members of the College of Surgeons.

VI. *Of the Bill in relation to the representation of the profession.* (p. 216.) No change is made in the Bill under this head. It is, however, to be hoped that, in the new Charters to be granted to the Colleges, some attempt will be made to institute a representation of the profession, the only kind of representation which, in our opinion, the profession ought to seek.

VII. *Of the Bill in relation to the government of the medical profession.* (p. 217.) *The Council of Health.* There is no change under this head. The objections we formerly made to the constitution of the Council of Health, therefore, still apply. It gratifies us, however, to find that the minister takes the same view of the proper mode of appointing the members advocated by us, though, having once proposed the present plan, he is unwilling to change it. We hope some alteration may be made before the Bill is finally passed. But should it be determined still to select members from the colleges and universities, we would suggest an important improvement in the mode of making the selection, viz. that instead of the regius professors being necessarily chosen, *ex officio*, the universities should be empowered to select *any* professor or member whom they might prefer as their representative. This seems, at first sight, an unimportant difference, but it can be shown to be of very great importance indeed, in the working of the council.

VIII. *Of the Bill in relation to the professional privileges and protection of medical practitioners.* (p. 222.) The changes under this head are the following:

1. The Apothecaries' Bill being only partially repealed, the power to prosecute unlicensed practitioners remains with the Company as before. As we contended, in our former article, that this power was really valuable to the profession—rather, however, *in terrorem* to intending or actual law-breakers, than from any actual exercise of it—we must regard its retention as, in so far, an advantage. But we are unable to appreciate the real amount of this advantage, because we are not informed as to the other relations of the Apothecaries' Company towards the members of the medical profession in future. As the general practitioner will be entirely independent of and unconnected with the Company—owing no allegiance, paying no fees to it—we cannot see what reason or grounds it can possess for prosecuting defaulters against a law which does not at all concern its interests; and we therefore fear the *terror* formerly attaching to it will be altogether lost upon evil-doers. We hope, however, from this virtual acknowledgment by the minister of the right to prosecute unlicensed practitioners, that, in the course of the discussion on the Bill, some means will be found to prevent this power of prosecution being a mere *brutum fulmen*, a word and nothing else! In short, we hope the old Apothecaries' Act will still be entirely repealed, and the power of prosecution placed elsewhere.

2. The only other alteration is the substitution of the second of the following clauses for the first.

*First Bill.* "And be it enacted, That every unregistered person who shall wilfully and falsely pretend to be, or take or use any name or title implying that he is registered under this Act, shall be deemed guilty of a misdemeanour in England and Ireland, and in Scotland of a crime and offence, and being convicted thereof, shall be punished by fine or imprisonment, or both, as the Court before which he shall be convicted shall award."

*Second Bill.* "And be it enacted, That every unregistered person who shall wilfully and falsely pretend to be, or take or use the name or title of Physician, Doctor, Bachelor or Inceptor in the Faculty of Medicine, or Surgeon or Licentiate in Medicine and Surgery, or Apothecary, or any name, title, or addition implying that he is registered under this Act, or recognized by law as a Medical or Surgical Practitioner, shall be deemed guilty," &c.

We do not pretend to be lawyers, but we fear that the original clause in the first Bill will be found, on trial, to be not one whit less or more stringent, as a law against irregular practitioners, than its more wordy successor. It certainly enlarges the list of names which it is penal to assume; but he must have a barren invention indeed, who could not go beyond the nomenclature of the new Bill.

On the whole, we may say, in conclusion, that the alterations made in the Bill in the new edition are for the better; but those who have done us the honour to read our former article need not be told that these alterations fall far short of our estimate of what is expedient.

---

